# Ginsenoside Rg2 alleviates myocardial fibrosis by regulating TGF-β1/Smad signalling pathway

**DOI:** 10.1080/13880209.2020.1867197

**Published:** 2021-02-03

**Authors:** Quanwei Wang, Wenwen Fu, Xiaofeng Yu, Huali Xu, Dayun Sui, Yeling Wang

**Affiliations:** a Departments of Cardiovascular Medicine, First Hospital, Jilin University, Changchun, PR China; b Department of Pharmacology, School of Pharmacy, Jilin University, Changchun, PR China

**Keywords:** Myocardial ischaemia, cardiac function, isoproterenol

## Abstract

**Context:**

*Panax ginseng* C.A. Meyer (Araliaceae) has cardioprotective effects. Ginsenosides are responsible for most of the pharmacological activities of ginseng.

**Objective:**

This study investigates the effect of ginsenoside Rg2 on myocardial fibrosis in myocardial ischaemia rats.

**Materials and methods:**

Male Wistar rats were divided into control, isoproterenol, ginsenoside Rg2 (5, 20 mg/kg) groups (*n* = 8). The rats were subcutaneously injected with isoproterenol (5 mg/kg) or normal saline (control group) once daily for 7 days. The animals were intragastrically treated with ginsenoside Rg2 or 0.5% CMC-Na (control and isoproterenol groups) daily for 28 days. At day 28, cardiac function, myocardial fibrosis, and TGF-β1/Smad signalling pathway were evaluated.

**Results:**

Compared with myocardial ischaemic rats, ginsenoside Rg2 at doses of 5, 20 mg/kg abated partially the augment of LVEDP (8.9 ± 1.3 vs. 7.5 ± 0.7, 7.2 ± 1.0 mmHg) and the decreases of the LVSP (96.75 ± 13.2 vs. 118.3 ± 19.4, 124.3 ± 21.3 mmHg), the + dp/dt (2142.8 ± 309.3 vs. 2598.6 ± 404.0, 2661.5 ± 445.2 mmHg/s), and the -dp/dt (1996.3 ± 306.3 vs. 2476.6 ± 289.7, 2509.6 ± 353.1 mmHg/s). Ginsenoside Rg2 (9.2 ± 0.9%, 8.5 ± 0.8%) alleviated myocardial fibrosis when compared with the isoproterenol group (10.1 ± 1.0%), which was accompanied by suppressed TGF-β1/Smad signalling in heart tissues.

**Conclusions:**

Ginsenosides from ginseng possess the property of alleviating myocardial fibrosis, improving cardiac function after myocardial ischaemia. Ginsenosides may be promising agents for improving the outcomes of patients with myocardial ischaemia.

## Introduction

Cardiovascular diseases (CVDs) are the primary cause of death worldwide (Hausenloy and Yellon 2013). Myocardial ischaemia, the most common CVDs, is caused by the reduction in coronary blood flow due to atherosclerotic coronary arterial obstruction. Researchers find that surgical approaches restoring blood flow of coronary arteries fail to improve cardiac function of patients with myocardial ischaemia (Ngu et al. 2018). On average, myocardial fibrosis will occur in 10% of patients. Myocardial fibrosis is the common cause of death after myocardial ischaemia, which accounts for a higher 5-year mortality (about 50%) (Benjamin et al. 2018). Myocardial fibrosis is characterised by the collagen formation, which leads to a chamber stiffness and an increased myocardial mass (Li et al. 2018a). Tremendous efforts have been made to develop therapeutics to attenuate myocardial fibrosis and improve cardiac function in patients of myocardial ischaemia. Traditional Chinese medicine (TCM) and small-molecule drugs are promising agents for preventing myocardial ischaemia-induced heart function impairment (Hashimoto et al. 2018).

In China, TCM has been used in treating CVDs for thousands of years. A great deal of interest has focussed on elucidating the mechanisms underlying the cardioprotective effects of the active ingredients of TCM (Fan et al. 2017). *Panax ginseng* C.A. Meyer (Araliaceae), a well-known TCM, is widely used for increasing vital energy and improving organ functions. Ginsenosides are the major active ingredients of ginseng. According to the structures of the extracted components of ginseng, ginsenosides are classified into three groups: the panaxadiol group including Rb1, Rb2, Rg3, and Rh2, the panaxatriol group including Re, Rh1, and Rg1, and oleanolic acid (Zhang et al. 2019). Recent work demonstrates that ginsenoside Rb1 can reduce the injury of myocardial ischaemia/reperfusion and then improve the heart function by inhibiting the apoptosis of cardiomyocytes (Li et al. 2020). Ginsenoside Rg1 exerts a protective effect against myocardial ischaemia-induced injury, which is associated with modulating energy metabolism and inhibiting myocardial apoptosis (Li et al. 2018b). Our previous studies indicate that ginsenoside Re treatment abrogates the myocardial fibrosis caused by myocardial ischaemia and then restores the heart function (Wang et al. 2019). Ginsenoside Rg2 is one of the cardinal components of ginseng. Ginsenoside Rg2 has a property of attenuating the neuronal damage induced by hypoxia in hippocampal neurons (Shuangyan et al. 2012). Ginsenoside Rg2 can also improve cerebral ischaemia-induced dementia through abating the apoptosis of neurons (Zhang et al. 2008). However, it is still not clear whether ginsenoside Rg2 has a protective effect against myocardial ischaemic injury. Therefore, this study investigates whether ginsenoside Rg2 could alleviate myocardial ischaemia-induced myocardial fibrosis and cardiac function impairment in rats.

## Materials and methods

### Ginsenoside Rg2

Ginsenoside Rg2 extracted from ginseng cultivated in China was kindly provided by Professor Yifa Zhou (School of Life Sciences, Northeast Normal University, Changchun, China). The purity of ginsenoside Rg2 was >98.0%.

### Animals

Male Wistar rats weighing 240–260 g were obtained from the Experiment Animal Centre of Jilin University (Changchun, China). Animals were housed on a 12 h light/dark cycle at a controlled temperature and humidity with unlimited access to food and water. The experiments were approved by the Ethics Committee of Jilin University (Approval number, JLU2018-03-0023) and were conducted according to the National Institutes of Health Guidelines for the Care and Use of Laboratory Animals (publication 86-23, revised in 1986).

### Experimental protocol

Experiment 1: Rats were randomly divided into two groups (*n* = 8): a control group (1 rat was excluded because of anaesthetic overdose) and a ginsenoside Rg2 at dose of 20 mg/kg group. The rats of the control group were intragastrically administered with 0.5% CMC-Na daily for 28 days. The animals of the ginsenoside Rg2 groups were intragastrically treated with ginsenoside Rg2 daily for 28 days. Experiment 2: Rats were randomly divided into four groups (*n* =8): a control group, an isoproterenol group and ginsenoside Rg2 (5, 20 mg/kg) groups. To induce myocardial fibrosis, the rats were injected subcutaneously with isoproterenol (Sigma-Aldrich, USA) at a dose of 5 mg/kg, once daily for 7 consecutive days (Zhang et al. 2014). Two hours later, the animals in the ginsenoside Rg2 groups were intragastrically treated with ginsenoside Rg2 at doses of 5 or 20 mg/kg, daily for 28 days. The rats of the control and the isoproterenol groups were intragastrically administered with 0.5% CMC-Na daily for 28 days.

### Cardiac function evaluation

At day 28, the body weights of the rats were recorded. The rats were anaesthetised by an intraperitoneal injection with a mixture of ketamine (80 mg/kg) and xylazine (10 mg/kg). A 2 F polyethylene catheter was inserted into the left ventricle through the right common carotid artery. The catheter was connected with a hemodynamic analysing system (Model RM-6000, Nihon Kohden, Japan). Then, the left ventricular systolic pressure (LVSP), left ventricular end diastolic pressure (LVEDP), and positive (+dp/dt) and negative (-dp/dt) maximal values of the first derivative of the left ventricular pressure were evaluated.

### Heart weight index assessment

The rats were sacrificed with overdoses of carbon dioxide after cardiac function evaluation. The hearts of the animals were collected and washed with PBS solution (4 °C). The aorta, atria, and adipose tissue were excised. Heart weights were weighed and heart weight index (mg/g) was calculated by dividing heart weight by body weight.

### Histopathological examination

The hearts of the rats were fixed with 10% formalin for 24 h. The specimens were embedded with paraffin and then were cut into 5 μm thick sections. According to the instruction of the manufacturer, the heart sections were stained with a Sirius red staining kit (Solarbio Life Sciences Company, China). Then, the histopathological examination was conducted by an experienced observer who was blinded to the experimental design under an Olympus microscope (IX-70, Olympus Corp., Japan). Ten fields were randomly selected from five sections in each heart. The ratio of the interstitial fibrosis area in the hearts was analysed and calculated with NIH-Image software (National Institutes of Health, Bethesda, MD, U.S.A.). The myocardial fibrosis was presented by the calculation: (fibrotic tissue area)/(fibrotic tissue area + cardiomyocyte area) × 100%.

### Western blot

Western blot was performed as previously described (Ahn et al. 2019). The membranes were incubated with the primary antibodies: rabbit anti-TGF-β1 (1:1000, Proteintech, USA), rabbit anti-α-smooth muscle actin (α-SMA) (1:2000, ImmunoWay Biotechnology, USA), rabbit anti-collagen I (1:2000, Abcam, UK), rabbit anti-Smad3 (1:1500, Abcam, UK), rabbit anti-p-Smad3 (1:1500, Abcam, UK) or rabbit anti-β-actin (1:1500, Beyotime Institute of Biotechnology, China) overnight at 4 °C. The membranes were processed with the horseradish peroxidase-labelled secondary antibody (1:2000, Beyotime Institute of Biotechnology, China). The bands were visualised using the ECL detection reagents (Beyotime Institute of Biotechnology, China). The ratio of the density of bands of the detected protein to that of β-actin was used for statistical analysis.

### Statistical analysis

Data were presented as the mean ± SD and processed with SPSS 22.0. Statistical analysis among various groups was conducted by one-way analysis of variance (ANOVA) with Tukey’s *post hoc* test. The *p* < 0.05 was considered as statistically significant.

## Results

### Effect of ginsenoside Rg2 on cardiac function and myocardial fibrosis in normal rats

**Figure 1.**
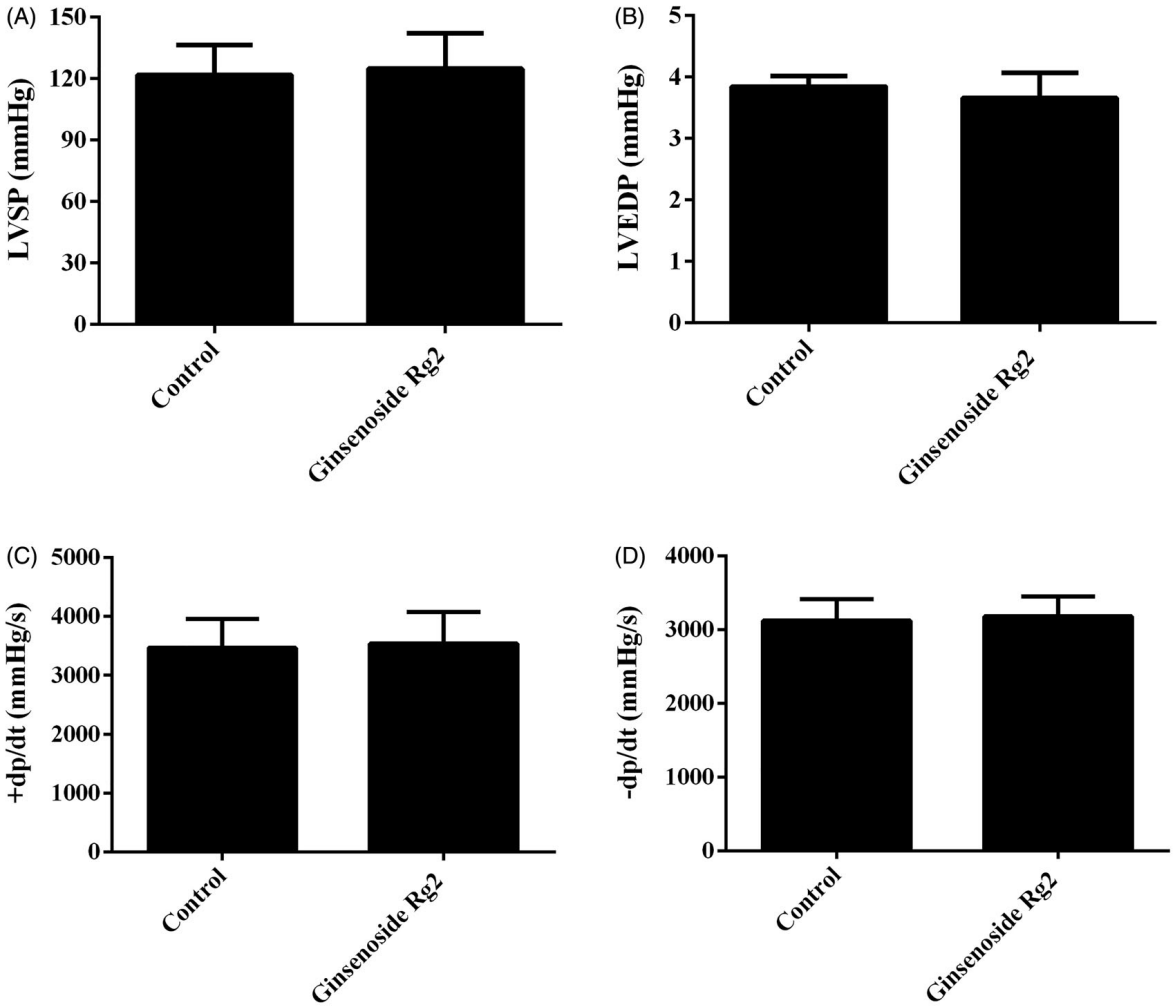
Effect of ginsenoside Rg2 on cardiac function in normal rats. Data are presented as the mean ± SD (*n* = 7–8).

**Figure 2.**
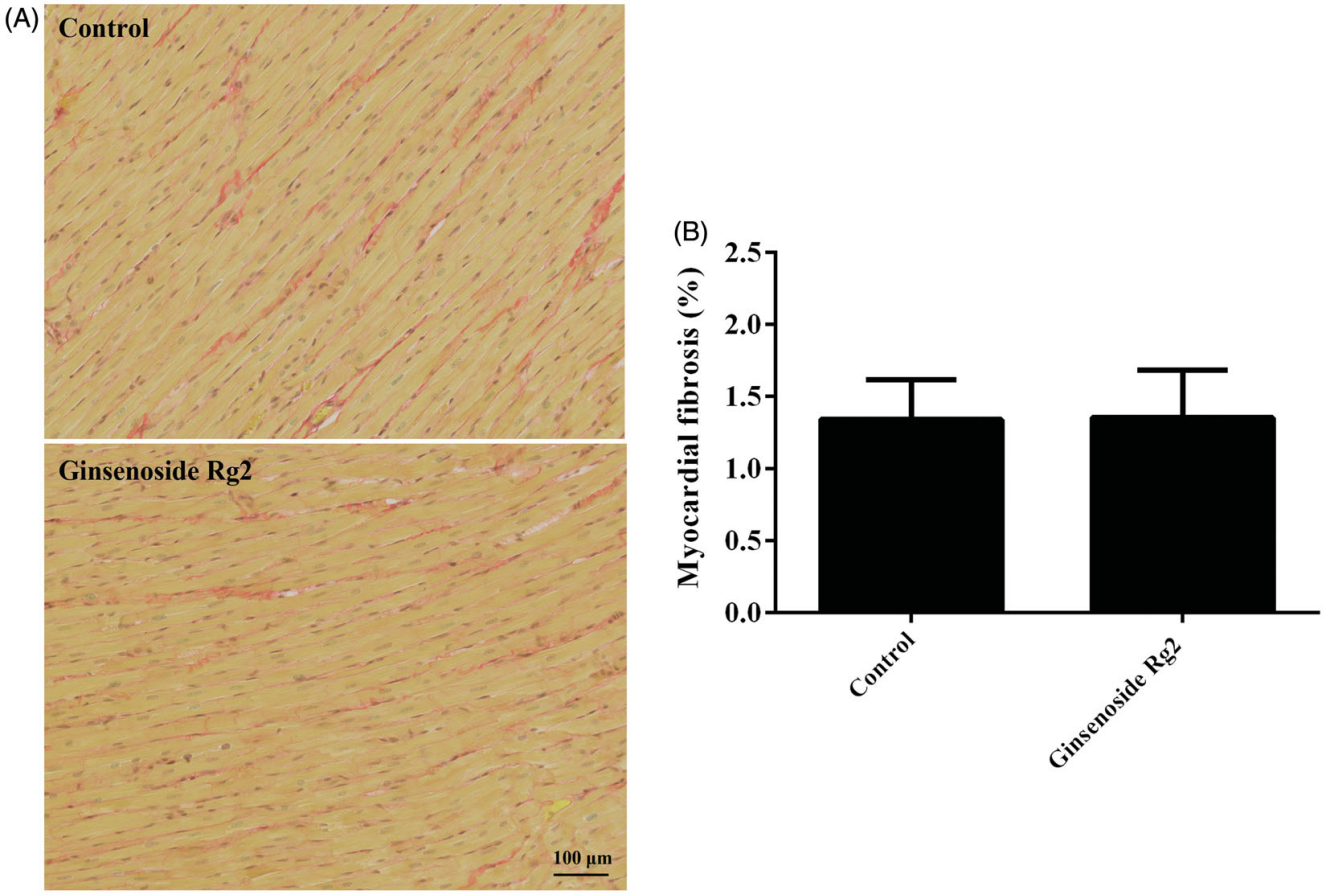
Effect of ginsenoside Rg2 on myocardial fibrosis in normal rats. Data are presented as the mean ± SD (*n* = 3). A indicates representative photomicrographs of Sirius red staining of the hearts. B indicates bar graph of myocardial fibrosis.

Compared with the control group, the LVSP, the LVEDP, the + dp/dt, and the -dp/dt of ginsenoside Rg2 group did not show significant changes (Figure 1, *p* > 0.05). Further histopathological examination demonstrated that myocardial fibrosis in the ginsenoside Rg2 group was the same as that of the control group (Figure 2, *p* > 0.05). These findings indicated that ginsenoside Rg2 alone had no effect on the cardiac function and the myocardial fibrosis in normal rats.

### Effect of ginsenoside Rg2 on cardiac function in myocardial ischaemic rats

**Figure 3.**
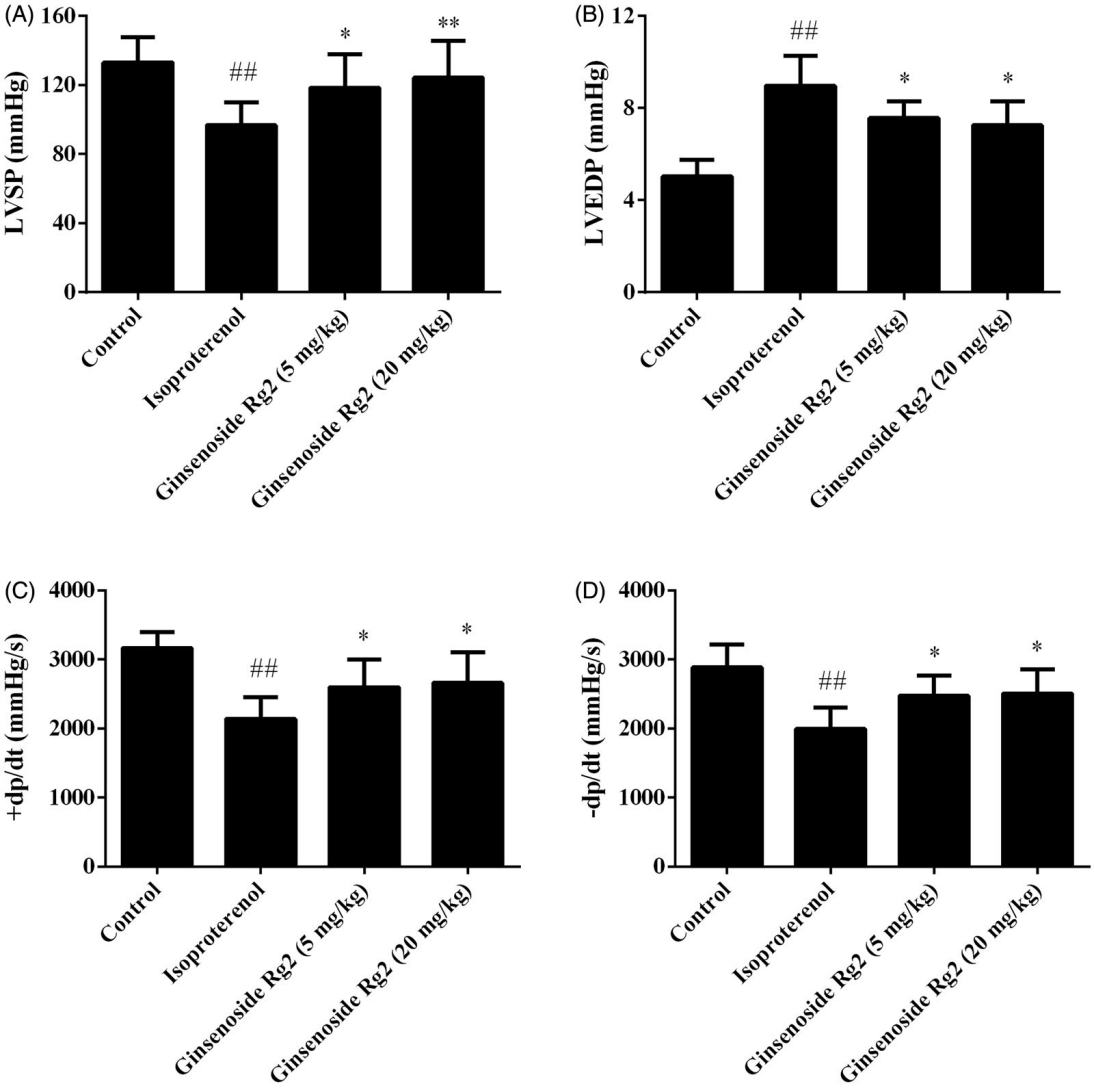
Effect of ginsenoside Rg2 on cardiac function in myocardial ischaemic rats. Data are presented as the mean ± SD (*n* = 8). ^##^*p* < 0.01 compared with control group; *p < 0.05, **p < 0.01 compared with isoproterenol group.

The cardiac function of the rats was evaluated with a hemodynamic analysing system. The LVEDP of the rats in the isoproterenol group was significantly increased, as compared with the control group (*p* < 0.01). The LVSP, the + dp/dt, and the –dp/dt in the isoproterenol group were markedly decreased (*p* < 0.01). Compared with the isoproterenol group, LVEDP of the rats in the ginsenoside Rg2 groups was significantly decreased, whereas the LVSP, the + dp/dt, and the –dp/dt of the ginsenoside Rg2 groups were increased (*p* < 0.05 or *p* < 0.01). These results demonstrated that ginsenoside Rg2 has a property improving the cardiac function in myocardial ischaemic rats (Figure 3).

### Effect of ginsenoside Rg2 on cardiac hypertrophy in myocardial ischaemic rats

**Figure 4.**
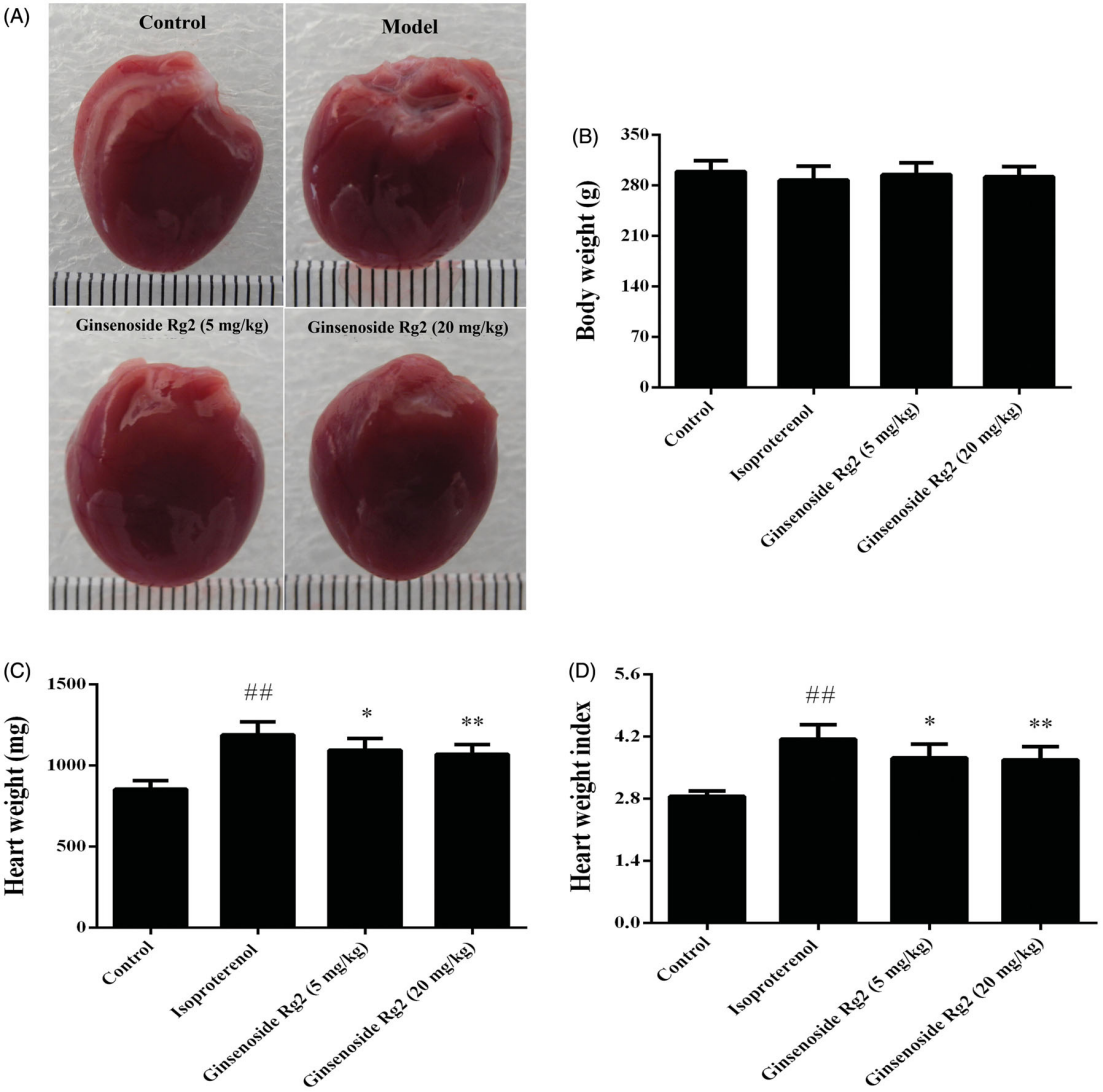
Effect of ginsenoside Rg2 on cardiac hypertrophy in myocardial ischaemic rats. Data are presented as the mean ± SD (*n* = 8). ^##^*p* < 0.01 compared with control group; *p < 0.05, **p < 0.01 compared with isoproterenol group. A indicates representative photomicrographs of the gross morphological hearts. B indicates bar graph of body weight. C indicates bar graph of heart weight. D indicates bar graph of heart weight index.

The gross morphological examination showed that hearts of the rats from the isoproterenol group were significantly larger than those from the control group (Figure 4(A)). In addition, heart weight and heart weight index were significantly increased in the isoproterenol group when compared to the control group (*p* < 0.01). Treatment with ginsenoside Rg2 at doses of 5, 20 mg/kg abrogated partially myocardial ischaemia-induced increases of heart weight and heart weight index (*p* < 0.05 or *p* < 0.01, Figure 4(B–D)).

### Effect of ginsenoside Rg2 on myocardial fibrosis in myocardial ischaemic rats

**Figure 5.**
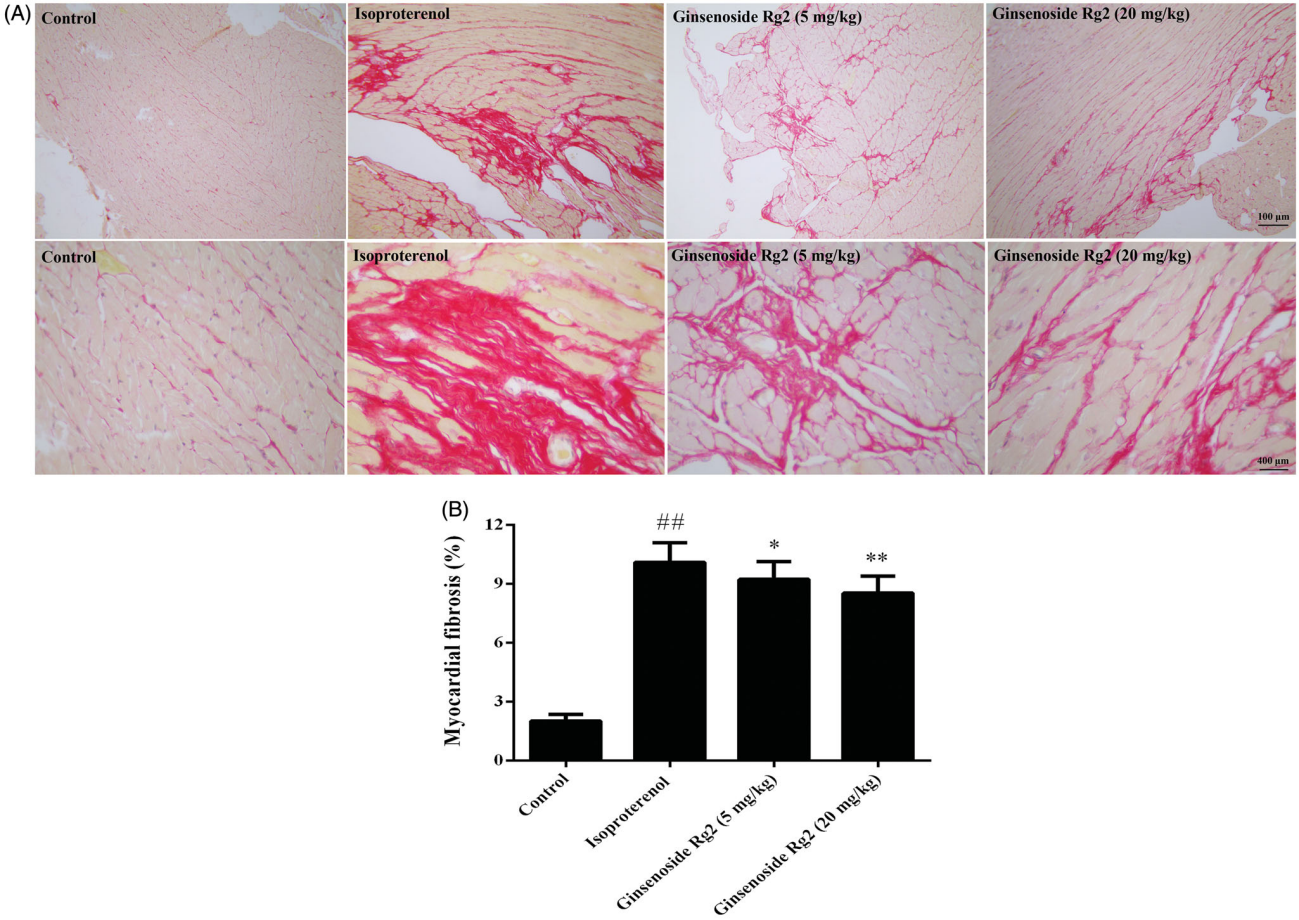
Effect of ginsenoside Rg2 on myocardial fibrosis in myocardial ischaemic rats. Data are presented as the mean ± SD (*n* = 3). ^##^*p* < 0.01 compared with control group; *p < 0.05, **p < 0.01 compared with isoproterenol group. A indicates representative photomicrographs of Sirius red staining of the hearts. B indicates bar graph of myocardial fibrosis.

The Sirius red staining showed that there was extensive collagen deposition in the border zone of the myocardial ischaemia in the myocardial ischaemic rats, whereas treatment with ginsenoside Rg2 at doses of 5, 20 mg/kg markedly reduced contents of collagen deposition in myocardial ischaemic rats (Figure 5(A)). The red-stained area was calculated as a percentage of the total area with Image-Pro Plus software. In line with the observations mentioned above, the red-appearing collagens of the heart tissues in myocardial ischaemic animals were significantly augmented (*p* < 0.01). However, ginsenoside Rg2 treatment markedly decreased the contents of the collagen content in the heart tissues (*p* < 0.05 or *p* < 0.01, Figure 5(B)).

### Effect of ginsenoside Rg2 on TGF-β1/Smad signalling pathway in myocardial ischaemic rats

**Figure 6.**
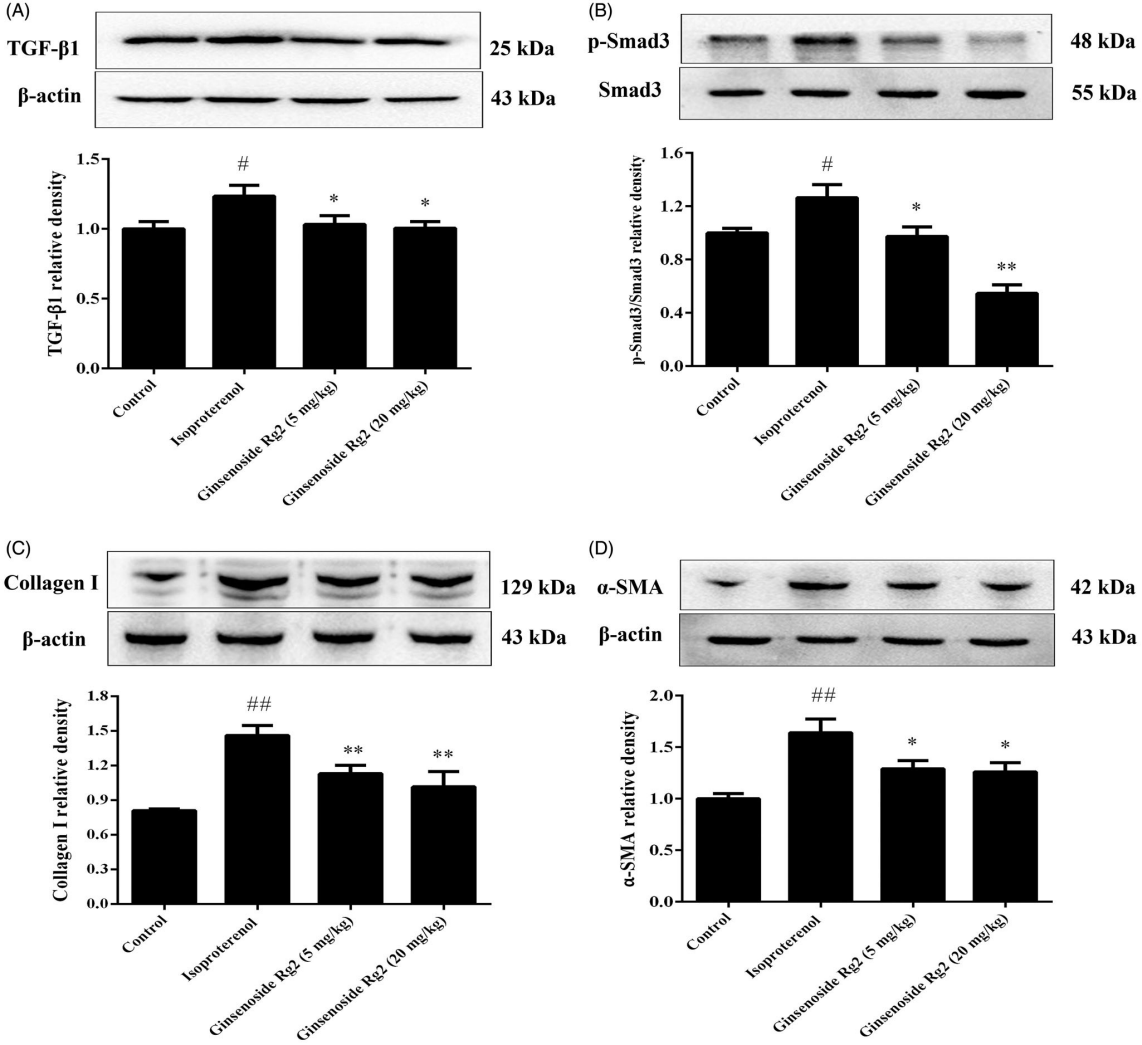
Effect of ginsenoside Rg2 on TGF-β1/Smad signalling pathway in myocardial ischaemic rats. Data are presented as the mean ± SD (*n* = 3). ^##^*p* < 0.01 compared with control group; *p < 0.05, **p < 0.01 compared with isoproterenol group. A indicates representative photograph and bar graph of TGF-β1. B indicates representative photograph and bar graph of p-Smad3. C indicates representative photograph and bar graph of collagen I. D indicates representative photograph and bar graph of α-SMA.

TGF-β1 leads to the production of collagens in myocardial fibroblasts. Smad3 is the downstream effectors of the TGF-β1 receptor. The effects of ginsenoside Rg2 on the TGF-β1/Smad signalling pathway in the heart tissues were investigated in the present study. The results showed that expression of TGF-β1, p-Smad3, collagen I and α-SMA were significantly up-regulated in the heart tissues of myocardial ischaemic rats (*p* < 0.05 or *p* < 0.01). However, treatment with ginsenoside Rg2 significantly decreased the expression of TGF-β1, p-Smad3, collagen I and α-SMA in the heart tissues (*p* < 0.05 or *p* < 0.01, Figure 6(A–D).

## Discussion

Cardiomyocytes death during myocardial ischaemia leads to a multiphase reparative response. The damaged heart tissues are replaced by a fibrotic scar produced by myofibroblasts. The progression of ischaemic necrosis induces interstitial and perivascular fibrosis in heart ventricular wall. The interstitial fibrosis and focal replacement scars can prevent cardiac rupture which is a fatal complication of myocardial ischaemia. However, the progressive myocardial fibrosis is detrimental as it produces abnormalities in ventricular function and leads to heart failure (Prabhu and Frangogiannis 2016). Therefore, attenuating myocardial fibrosis would offer a favourable prognosis in patients with ischaemic heart disease. The present study demonstrated for the first time ginsenoside Rg2 alone did not induce myocardial fibrosis and had no adverse effect on cardiac function. Ginsenoside Rg2 showed a property of alleviating the myocardial fibrosis and improving the impairment of cardiac function by regulating the TGF-β1/Smad signalling pathway after myocardial ischaemia.

Myocardial ischaemia causes the changes in the size, shape, and function of the heart and leads to cardiac failure. It is well known that the heart has no capacity of regeneration, and the lost cardiomyocytes will be replaced by a fibrotic scar (Laflamme and Murry 2005). The fibrotic process after myocardial ischaemia is classified into replacement and reactive fibrosis, both of which are associated with myofibroblasts (Gao et al. 2019). The characteristic of myofibroblasts is the expression of α-SMA. Therefore, the content of α-SMA is a commonly used to evaluate the myofibroblasts in heart tissues (Shinde et al. 2017). Collagen I is the primary structural protein in the heart interstitium and is synthesised by myofibroblasts (van den Borne et al. 2010). Myofibroblasts secrete a great deal of interstitial collagens after myocardial ischaemia, which is crucial for increasing heart weight and impairing cardiac function. The current study showed that cardiac function was impaired after myocardial ischaemia and there were significant depositions of collagen I in the heart tissues. However, treatment with ginsenoside Rg2 for 28 days improved cardiac function and decreased collagen I depositions, suggesting that ginsenoside Rg2 can ameliorate ischaemia-induced myocardial fibrosis.

Numerous factors play a role in modulating the fibrosis induced by myocardial ischaemia. TGF-β1 is the cardinal pro-fibrotic growth factor involved in fibroblast activation and collagens production (Dobaczewski et al. 2011). The previous report demonstrates that TGF-β1 leads to the transdifferentiation of myofibroblast and therefore increases the synthesis of extracellular matrix protein (Dobaczewski et al. 2012). In hearts, TGF-β1 will be rapidly produced and released in response to ischaemia. TGF-β1 exerts its effects through binding to its receptor and induces phosphorylation of Smads. Smad3 is one of the major downstream regulators that are associated with TGF-β1-mediated myocardial fibrosis. The phosphorylation of Smad3 forms heteromeric complexes with Smad4 and then they translocate into the nucleus. In the nucleus, the complex regulates the expression of target genes related to fibrosis (e.g., collagen I) after binding to the Smad-binding element (Shi and Massagué 2003). In this study, we found that the expression of collagen I in the myocardial ischaemic rat hearts was increased. Also, expressions of TGF-β1 and p-Smad3 were augmented, suggesting that the TGF-β1/Smad3 signalling pathway was involved in the process of myocardial fibrosis. Consistent with our hypothesis, ginsenoside Rg2 reduced expressions of TGF-β1, p-Smad3, and collagen I. These findings suggested that ginsenoside Rg2 alleviates myocardial fibrosis and therefore improves the impairment of cardiac function by regulating TGF-β1/Smad signalling pathway after myocardial ischaemia.

## Conclusions

The present experiments demonstrated that ginsenoside Rg2 possesses the property of alleviating myocardial fibrosis and improving cardiac function by regulating the TGF-β1/Smad signalling pathway after myocardial ischaemia. This study provides insights into the mechanisms of ginseng in the clinical application for myocardial ischaemia.

## Geolocation information

Changchun is at 125.35 degrees East longitude and 43.88 degrees North latitude.
